# Optimal Learning Samples for Two-Constant Kubelka-Munk Theory to Match the Color of Pre-colored Fiber Blends

**DOI:** 10.3389/fnins.2022.945454

**Published:** 2022-07-01

**Authors:** Junfeng Li, Dehong Xie, Miaoxin Li, Shiwei Liu, Chun’Ao Wei

**Affiliations:** ^1^School of Packaging and Printing Engineering, Henan University of Animal Husbandry and Economy, Zhengzhou, China; ^2^College of Information Science and Technology, Nanjing Forestry University, Nanjing, China

**Keywords:** color matching, pre-colored fiber, Kubelka-Munk theory, mixing theory, fabric, absorption coefficient, scattering coefficient

## Abstract

Due to the dyeing process, learning samples used for color prediction of pre-colored fiber blends should be re-prepared once the batches of the fiber change. The preparation of the sample is time-consuming and leads to manpower and material waste. The two-constant Kubelka-Munk theory is selected in this article to investigate the feasibility to minimize and optimize the learning samples for the theory since it has the highest prediction accuracy and moderate learning sample size requirement among all the color prediction models. Results show that two samples, namely, a masstone obtained by 100% pre-colored fiber and a tint mixed by 40% pre-colored fiber and 60% white fiber, are enough to determine the absorption and scattering coefficients of a pre-colored fiber. In addition, the optimal sample for the single-constant Kubelka-Munk theory is also explored.

## Introduction

In the textile industry, blending two or more pre-colored fibers to produce a variety of colors is an important coloring method, which has special color perception effects such as structural non-uniform or mottled appearance (Jolly et al., 2021). One of the most important tasks for the industry is to find the appropriate proportion of the pre-colored fibers to exactly match the color of a target sample required by the customer. To solve this problem, a color prediction model is required to describe the relationship of the spectral reflectance between a fabric and its individual pre-colored fiber. Assuming that *R*_λ_ is the spectral reflectance of the fabric at wavelength λ, and *R*_*i*_,_λ_ (*i* = 1,2,… ,*n*) is the spectral reflectance of the *i*th pre-colored fiber that composes the fabric at fractional concentration *c*_*i*_ (*c*_*i*_ ≥ 0 and $\sum_{i=1}^{n}c_{i}=1$), the general purpose of a color prediction model can be formulated as follows:


(1) $$R_{\lambda }=\text{F}\left(c_{1},c_{2},\ldots ,c_{n},R_{1,\lambda },R_{2,\lambda },\ldots ,R_{n,\lambda }\right)$$

It is obvious that given the spectral reflectance and the corresponding fractional concentration of the pre-colored fibers, the mapping function F aims to predict the color of the fabric obtained by mixing the fibers. In turn, the fractional concentration can be evaluated by the inverse of the mapping function according to the spectral reflectance of the fabric and the pre-colored fibers that compose the fabric.

Depending on the mechanism that constructs the mapping function, the color prediction model can be divided into three types, namely, empirical model, physical model, and artificial intelligence model. The most direct and simplest empirical model regards the mapping (blending) process as a linear function (system) (Hemingray and Westland, 2016). The spectral reflectance of the fabric is a linear combination of the spectral reflectance of the pre-colored fibers weighted by the corresponding fractional concentration. That is,


(2) $$R_{\lambda }=\sum_{i=1}^{n}c_{i}R_{i,\lambda }$$

It has been proved that the performance of Eq. 2 is poor in practice. To improve the performance, many efforts have been devoted to finding new functions that map the spectral reflectance into new space in which the mapped data are additive. The main idea of these improvements is to seek the mapping functions that satisfy the following equation:


(3) $$\text{F}\left(R_{\lambda }\right)=\sum_{i=1}^{n}c_{i}\text{F}\left(R_{i,\lambda }\right)$$

Several empirical mapping functions have been reported in the literature so far to accommodate Eq. 3 (Aldeeson et al., 1961; Love et al., 1965; Minato, 1977). To the best of our knowledge, the most representative ones for predicting the color of fiber blends are the Stearns-Noechel function (Stearns and Noechel, 1944; Rong et al., 2007) and the Friele function (Friele, 1952; Philips-Invernizzi et al., 2002a). The Stearns-Noechel function (Stearns and Noechel, 1944) has the following form:


(4) $$\text{F}\left(R_{\lambda }\right)=\frac{1-R_{\lambda }}{b\left(R_{\lambda }-0.01\right)+0.01}$$

where *b* is an empirical constant for the function. Subsequent studies on the function have revealed that the constant varies with fiber types and wavelengths (Philips-Invernizzi et al., 2002b; Rong and Feng, 2006, Sabir, 2011). It should be determined experimentally again once another type of fiber is used to blend the fabric that limits the universality and practicality of the function.

The Friele function (Friele, 1952) can be expressed as follows:


(5) $$\text{F}\left(R_{\lambda }\right)=e^{-\sigma {\left(1-R_{\lambda }\right)}^{2}/\left(2R_{\lambda }\right)}$$

where σ is the Friele parameter. This function has a similar drawback with the Stearns-Noechel function since the Friele parameter also varies with fiber types and should be redetermined experimentally (Miller et al., 1963, Philips-Invernizzi et al., 2002a). Although previous studies have recommended several values for different fiber types, the parameters should be redetermined experimentally for the same fiber type as producing regions, harvesting methods, and growing environments influence the quality of the fiber and then lead to the variation of the parameter.

The most commonly used physical model in color prediction of fiber blends is the single-constant and two-constant Kubelka-Munk (K-M) theory (Burlone, 1983, 2007; Walowit et al., 1988; Amirshahi and Pailthorpe, 1994). The theory includes two parts, namely, the K-M theory (Kubelka and Munk, 1931; Kubelka, 1948, 1954) and the mixing theory, proposed by Duncan (1949). The K-M theory is a special solution to the general radiative transfer problem that characterizes the radiance of light propagating inside a layer (Yang and Kruse, 2004). It maps the spectral reflectance of the fabric into the absorption and scattering characteristics of the fabric. For an opaque sample, the K-M theory is formulated as follows:


(6) $$\text{F}\left(R_{\lambda }\right)={\left(\frac{K}{S}\right)}_{\lambda }=\frac{{\left(1-R_{\lambda }\right)}^{2}}{2R_{\lambda }}$$

where *K*_λ_ is the absorption coefficient and *S*_λ_ is the scattering coefficient of the sample. Although they are the optical characteristics of the fabric, they cannot be measured directly. Therefore, they are estimated from the spectral reflectance of the fabric.

The mixing theory describes the relationship of absorption and scattering coefficients between a fabric and its individual fiber. It assumes that the absorption and scattering coefficients of each fiber are additive when weighted by the corresponding fractional concentration, and the sum is the absorption coefficient *K*_λ_ and scattering coefficient *S*_λ_ of the fabric blended by the fibers. That is,


(7) $$K_{\lambda }=\sum_{i=1}^{n}c_{i}k_{i,\lambda }$$

(8) $$S_{\lambda }=\sum_{i=1}^{n}c_{i}s_{i,\lambda }$$

where *k*_*i*,λ_ and *s*_*i*,λ_ are the absorption and scattering coefficients of the *i*th fiber that composes the fabric. Eqs 7, 8 together with K-M theory are known as the two-constant K-M theory since it contains two independent parameters, *k*_λ_ and *s*_λ_ , for each pre-colored fiber. The ratio between absorption and scattering coefficients of the fabric can be calculated by Eq. 7 divided by Eq. 8 as follows:


(9) $${\left(\frac{K}{S}\right)}_{\lambda }=\frac{\sum_{i=1}^{n}c_{i}k_{i,\lambda }}{\sum_{i=1}^{n}c_{i}s_{i,\lambda }}$$

Under the condition that the fibers composing the fabric have a similar scattering ability, Eq. 9 can be further simplified as follows:


(10) $${\left(\frac{K}{S}\right)}_{\lambda }=\frac{\sum_{i=1}^{n}c_{i}k_{i,\lambda }}{s_{\lambda }}=\sum_{i=1}^{n}c_{i}{\left(\frac{k}{s}\right)}_{i,\lambda }$$

It indicates that the absorption and scattering coefficient ratio of the fabric can be a linear combination of those of the fibers composing the fabric when the fibers have a similar scattering ability. Equation 10 together with the K-M theory is referred as the single-constant K-M theory since it only includes one independent parameter, (*k*/*s*)_λ_ , for each pre-colored fiber.

During the past several decades, great progress has been made in the field of artificial intelligence techniques. Artificial neural network (ANN) model, as an important branch of artificial intelligence, has been widely used in textile engineering in recent years to predict the color changes that take place after certain production processes (Shamsnateri et al., 2006; Shamey and Hussain, 2008; Furferi and Carfagni, 2010b; Almodarresi et al., 2013; Hwang et al., 2015; Jawahar et al., 2015, Kan and Song, 2015). ANN is an information processing system that simulates the structure and function of the human brain. A learning process is required to train the values of the weights and biases of the network. It works as a black box, producing outputs according to the inputs it receives with powerful processing capability and non-linear mapping properties. Furferi and Governi (2011) first applied ANN to the color prediction of pre-colored fiber blends and concluded that the prediction results are reliable. Then, they also combined the K-M theory with ANN to implement the color prediction of fiber blends (Furferi et al., 2016). The non-linear mapping properties of ANN bypassed the linear additive assumption that derives Eq. 10 in single-constant K-M theory which enhanced the performance of single-constant K-M theory. Shen et al. (2016) also combined the Stearns-Noechel model with ANN to enhance the performance of Stearns-Noechel model. Hemingray and Westland (2016) proposed a novel approach to using ANN to predict the color of fiber blends where rather than using a single network, a set of small neural networks was used, each of which predicted reflectance at a single wavelength. The results showed that the novel approach is more robust than the conventional approach when the number of training examples was small. In general, all these studies indicate that ANN can be used for the color prediction of pre-colored fiber blends, but it requires adequate samples for learning; otherwise, its results are poor.

Comparisons of these models have also appeared in the literature in recent years (Furferi and Carfagni, 2010a; Hemingray and Westland, 2016). Taken together, it can be concluded that the ANN model has the highest prediction accuracy when the learning samples are adequate; the two-constant K-M theory comes second; then comes the Stearns-Noechel model and the Friele model; and the single-constant K-M theory has the worst accuracy. In terms of prediction accuracy, the ANN model and the two-constant K-M theory are more suitable for the color prediction of pre-colored fiber blends since their average color difference is usually less than the threshold value of 0.8 set for quality inspection. Due to the dyeing process, even though the fibers are dyed in the same equipment with the same dye, their color, however, can vary significantly if they belong to different batches. This means that the optical characteristics of the dyed fibers are variable, and new learning samples are required to be prepared to train the parameters in the prediction models. Yet, the preparation process is time-consuming and also leads to manpower and material waste (Furferi et al., 2015). To find the appropriate proportion of the pre-colored fiber for a fabric, a skilled technician only needs about six rounds of adjusting. Thus, the learning samples required by the model should be as less as possible. As for the ANN model and the two-constant K-M theory that can meet the accuracy requirement for practical production, if *n* pre-colored fibers are used to obtain the fabric, to match the color of the fabric, the number of learning samples required by the ANN model is massive, while the traditional two-constant K-M theory needs *n* ladders, each of which includes several samples obtained by mixing the white (or black) fiber and the pre-colored fiber at different fractional concentrations.

Based on this point of view, the two-constant K-M theory is selected in this study to investigate the feasibility to minimize and optimize the learning samples for the theory. The single-constant K-M theory is also explored to reveal the mechanism that leads to the prediction error. The minimum samples required by the theories to determine the parameters are analyzed, and these samples are compared to find the optimal learning samples according to the revealed mechanism.

## Experiment

### Materials

Cotton fibers (1.67 dtex and average 37 mm long) dyed by four reactive dyes are selected in this work as pre-colored fibers. Together with the raw undyed fibers, a total of five fibers (White 01, Green 09, Blue 72, Red 18, and Yellow 03) are used. First, the fibers are fed to a carding machine three times to obtain homogeneous mixtures. Second, the mixtures are spun into yarns using open-end spinning with a count of 29.2 tex and a twist coefficient of 450 atex. Then, the yarns were knitted into single jersey fabrics with 24 threads/inch as experimental samples. Two sets of samples are prepared in this study. Set A has 22 samples mixed by three pre-colored fibers, namely, White 01, Green 09, and Blue 72. Green 09 and Blue 72 are mixed with White 01 individually in fractional concentration increments of 20%, respectively, to prepare 11 ladder samples. Other samples are prepared by 7 binary and 4 ternary mixtures of these pre-colored fibers. This set is used to analyze the feasibility to optimize the learning samples for the theory.

Another Set B including 50 samples mixed by all the five fibers is used to test the validity of the proposed methods. Eight samples are mixed according to the conclusion drawn from the first set, namely, Green 09, Blue 72, Red 18, and Yellow 03 that are mixed with White 01 in fractional concentrations of 40%:60% and 60%:40%. The other samples are mixed by randomly mixing these five pre-colored fibers. There are 10 binary mixtures, 18 ternary mixtures, and 9 quaternary mixtures besides five pure samples with 100% fractional concentration.

### Measurement

After spinning and weaving into knitted fabrics, the spectral reflectance of the samples was measured by the Ci7800 benchtop sphere spectrophotometer. The measurement geometry was d/8°, and the measurement aperture used was 25 mm. The original spectral data were measured at 10 nm intervals within the range of 360–750 nm. To reduce the potential measure error, each sample was measured three times, and the average was calculated as the measure result. The wavelength range of 400–700 nm was taken providing the 31-dimensional spectral data at last to implement the color prediction.

The K-M theory does not take the discontinuity of the refractive index existing in the interface between air and fabric into account. A correction to the measured spectral reflectance is necessary before inducing the measured spectral reflectance into the theory. The equation used for the correction is well-known as the Saunderson correction (Saunderson, 1942). Assuming that collimated light from the air strikes the fabric, a portion *r*_1_ is reflected back to the air. In turn, a portion *r*_2_ is reflected back when the light enters the interface from the fabric. After infinite internal reflection, the relationship between the measured spectral reflectance *R*_*m*,λ_ and the corrected spectral reflectance *R*_*c*,λ_ can be represented as follows:


(11) $$R_{c,\lambda }=\frac{R_{m,\lambda }-\alpha k_{1}}{1-k_{1}-k_{2}\left(1-R_{m,\lambda }\right)}$$

where α is the adjustable factor that changes with the measuring geometry. For measuring with specular component included, α = 1. Otherwise, α = 0. α = 0 is adopted in this study to implement the correction since the samples are measured by d/8° measurement geometry with specular components excluded. According to our experience, *k*_1_ = 0.08 and *k*_2_ = 0.5 are utilized to implement the correction.

### Evaluation

Three metrics are adopted to evaluate the accuracy of the prediction results. The root mean squared error (RMSE) between the predicted and targeted spectral reflectance is selected as the spectral metric. The CIEDE2000 color difference Δ*E*_00_ under the CIE standard illuminant D65 and the CIE 1931 standard observer is calculated as the colorimetric metric (Penacchio et al., 2021). Influenced by the factors such as weighting precision and mixture homogeneity of the pre-colored fibers, the predicted and targeted concentrations often differ. Thus, the Euclidean distance between the predicted and targeted fractional concentrations is adopted as the concentration error (CE) metric.

## Materials and Methods

### Determination of Absorption and Scattering Coefficient Ratio

The single-constant K-M theory only involves the absorption and scattering coefficient ratio of the pre-colored fibers. The ratio can be calculated by Eq. 6 with the corrected spectral reflectance of its masstone (pure sample with 100% fractional concentration). Due to the intense absorption ability of the masstone obtained by the pure pre-colored fiber, its spectral reflectance is usually very small. However, the direct use of the sample will result in a higher color prediction error since the measure noise has a relatively significant influence on the small spectral data. Thus, the tint mixed by the pre-colored fiber and the white fiber is utilized in practice to reduce the influence of the measure noise and then to improve the accuracy of the calculated ratio. Given the ratio (*k*/*s*)_*w*,λ_ of the white fiber, according to Eq. 10, the ratio (*K*/*S*)_*tint*,λ_ of a tint can be expressed as follows:


(12) $${\left(\frac{K}{S}\right)}_{tint,\lambda }=c\cdot {\left(\frac{k}{s}\right)}_{\lambda }+\left(1-c\right){\left(\frac{k}{s}\right)}_{w,\lambda }$$

where (*k*/*s*)_λ_ is the absorption and scattering coefficient ratio of the pre-colored fiber; *c* is its fractional concentration in the tint; subscript *w* and *tint* represent the white and the tint, respectively. The traditional methods usually prepare several tints and use the least square regression method to solve the absorption and scattering coefficient ratio of the pre-colored fiber. In this study, only one tint sample is used, and then, the ratio can be calculated by:


(13) $${\left(\frac{k}{s}\right)}_{\lambda }=\frac{{\left(K/S\right)}_{tint,\lambda }-\left(1-c\right){\left(k/s\right)}_{w,\lambda }}{c}$$

**FIGURE 1:**
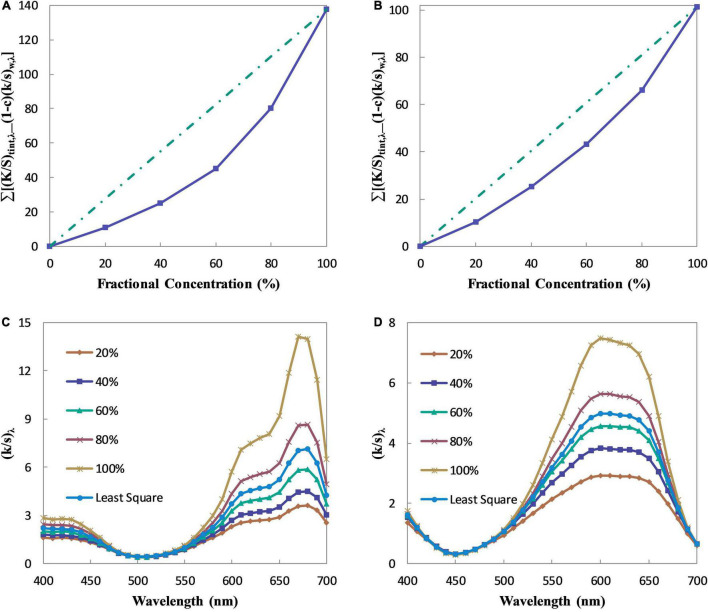
The absorption and scattering coefficient ratios of Green 09 and Blue 72. **(A)** The relationship between optical property and fractional concentration of Green 09. **(B)** The relationship between optical property and fractional concentration of Blue 72. **(C)** The absorption and scattering coefficient ratios of Green 09. **(D)** The absorption and scattering coefficient ratios of Blue 72.

The numerator and denominator in Eq. 13 should keep a linear relationship in theory because the absorption and scattering coefficient ratio of the pre-colored fiber is constant (Völz, 2002). In fact, the linear relationship, however, is not strictly obeyed. As shown in Figures 1A,B, their relationships are always a concave curve. This phenomenon has also been found in other industries dealing with colorants such as paints (Berns and Mahnaz, 2007) and printing ink (Berns, 1993), while a convex curve has been found in dyes’ color prediction (Yang et al., 2009) since the means of its vertical and horizontal coordinates are different from that here. Figures 1C,D is the absorption and scattering coefficient ratios of Green 09 and Blue 72 calculated by different tints. With the increase of the pre-colored fiber, the calculated ratio increases. It indicates that the absorption ability of the fabric is enhanced with the addition of the pre-colored fiber, and this is the reason that leads to the non-linear relationship in Figures 1A,B. It can be inferred that the tint that is selected to determine the ratio has influence on the color prediction accuracy. On the contrary, the absorption and scattering coefficient ratio calculated by the least square regression method lies between those calculated by the 60% tint and the 80% tint. In another words, an optimal tint exists in the color matching of pre-colored fiber blends, and the optimal tint is the tint with about 80% fractional concentration of the pre-colored fiber. Thus, a tint mixed by 80% pre-colored fiber and 20% white fiber is enough to determine the absorption and scattering coefficient ratio of pre-colored fiber if the ratio of the white fiber is pre-known.

### Determination of Absorption and Scattering Coefficients

Due to the weak absorption and intense scattering ability, the scattering coefficient of the white fiber can be assumed as a unit at all wavelengths. Then, the absorption coefficient of the white fiber can be determined as (*k*/*s*)_*w*,λ_ by applying Eq. 6. As for the pre-colored fiber, Eq. 9 can be transposed and collected as follows:


(14) $${\left(\frac{K}{S}\right)}_{\lambda }\cdot s_{\lambda }-k_{\lambda }=\frac{1-c}{c}\cdot \left[{\left(\frac{k}{s}\right)}_{w,\lambda }-{\left(\frac{K}{S}\right)}_{\lambda }\right]$$

where *k*_λ_ and *s*_λ_ are the absorption and scattering coefficients of the pre-colored fiber. To determine these two unknowns, at least two samples are required. The traditional methods usually prepare several tints and use the least square regression method to solve the absorption and scattering coefficients. In this study, two samples are selected to construct the simultaneous equations to solve the unknowns. The masstone is chosen as one of the samples because it can be easily prepared without weighting and mixture. Another sample can only be a tint mixed by the pre-colored fiber and the white fiber. The simultaneous equations become:


(15) $$\left\{ \begin{array}{c} {\left(\frac{K}{S}\right)}_{masstone,\lambda }\cdot s_{\lambda }-k_{\lambda }=0\\ {\left(\frac{K}{S}\right)}_{tint,\lambda }\cdot s_{\lambda }-k_{\lambda }=\frac{1-c}{c}\cdot \left[{\left(\frac{k}{s}\right)}_{w,\lambda }-{\left(\frac{K}{S}\right)}_{tint,\lambda }\right] \end{array}\right.$$

Then, the absorption and scattering coefficients of the pre-colored fiber can be calculated as follows:


(16) $$s_{\lambda }=\frac{1}{c}\cdot \left\{\left(1-c\right)\cdot \left[{\left(\frac{k}{s}\right)}_{w,\lambda }-{\left(\frac{K}{S}\right)}_{tint,\lambda }\right]/\left[{\left(\frac{K}{S}\right)}_{tint,\lambda }-{\left(\frac{K}{S}\right)}_{masstone,\lambda }\right]\right\}$$

(17) $$k_{\lambda }=\frac{1}{c}\cdot \left\{\left(1-c\right)\cdot {\left(\frac{K}{S}\right)}_{masstone,\lambda }\cdot \left[{\left(\frac{k}{s}\right)}_{w,\lambda }-{\left(\frac{K}{S}\right)}_{tint,\lambda }\right]/\left[{\left(\frac{K}{S}\right)}_{tint,\lambda }-{\left(\frac{K}{S}\right)}_{masstone,\lambda }\right]\right\}$$

**FIGURE 2:**
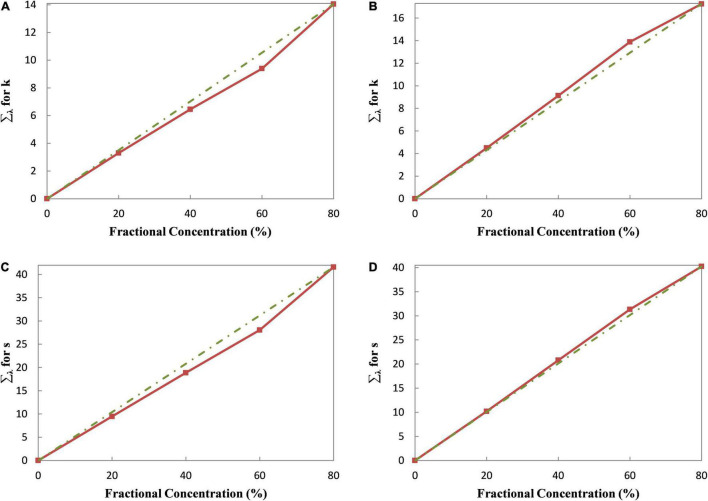
The relationships between optical property and fractional concentration. **(A)** The relationship for absorption coefficients of Green 09. **(B)** The relationship for absorption coefficients of Blue 72. **(C)** The relationship for scattering coefficients of Green 09. **(D)** The relationship for scattering coefficients of Blue 72.

**FIGURE 3:**
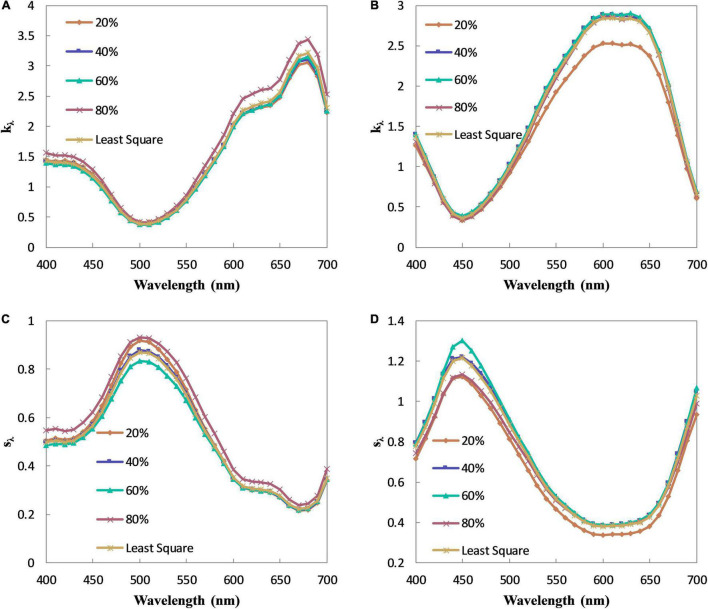
The absorption and scattering coefficients of Green 09 and Blue 72. **(A)** The absorption coefficients of Green 09. **(B)** The absorption coefficients of Blue 72. **(C)** The scattering coefficients of Green 09. **(D)** The scattering coefficients of Blue 72.

The relationship between the terms in the braces and the fractional concentration in Eqs 16, 17 should be linear in theory since the absorption and scattering coefficients of the pre-colored fiber are constant. As analyzed above, the linear relationship, however, is not strictly obeyed in practice. Figures 2A–D shows the relationships of Green 09 and Blue 72. Compared with Figures 1A,B, the relationships in Figures 2A–D become more linear. It means that the two-single K-M theory has better color prediction accuracy than the single-single K-M theory. Besides, the tint that is selected in the two-single K-M theory has less influence than that in the single-single K-M theory. On the contrary, with the use of the masstone samples, the curve shapes between two different fibers may be opposite [i.e., (a) vs. (b); (c) vs. (d)], whereas the curve shapes of the same fiber are similar [i.e., (a) and (c); (b) and (d)]. An optimal tint may exist here for the two-constant K-M theory. The absorption and scattering coefficients of Green 09 and Blue 72 calculated by different tints are shown in Figures 3A–D. It can be inferred that the optimal tint is the tint with about 40% fractional concentration of the pre-colored fiber.

## Results and Discussion

The aim of color matching is to find the concentrations that minimize the difference between the predicted and targeted spectral reflectance from optical constants. According to the methods to evaluate the difference, the optimization objective of the color matching can be divided into two types: colorimetric matching and spectrophotometric matching. The optimization objective of colorimetric matching is to minimize the color difference between the predicted and targeted samples. It can be expressed as follows:


(18) $$\min f\left(c_{1},c_{2},..,c_{n}\right)={\left[\sum_{i=1}^{3}{\left(T_{predicted,i}-T_{targeted,i}\right)}^{2}\right]}^{1/2}$$

where *T*_*i*_ (*i* = 1,2,3) is the tristimulus of the samples; the fractional concentrations need to satisfy the constraint that *c_i_* ≥ 0 and $\sum_{i=1}^{n}c_{i}=1$. The corresponding linear iterative algorithms that solve this problem have also been proposed in the literature (Allen, 1974; Amirshahi et al., 1995; Karbasi et al., 2008). Moreover, a new matching strategy based on the equalization of the first three principal component coordinates of the predicted sample and targeted sample in a 3D eigenvector space has been reported in recent years (Agahian and Amirshahi, 2008; Shams-Nateri, 2009; Mohtasham et al., 2012). Although it shows a better performance than the colorimetric matching in terms of spectral and colorimetric accuracy, its matching principle and optimization procedure are similar to that of the colorimetric matching. The optimization objective of spectrophotometric color prediction is to minimize the RMSE of the spectral reflectance between the predicted and targeted samples. That is,


(19) $$\min f\left(c_{1},c_{2},..,c_{n}\right)={\left[\sum_{\lambda }{\left(R_{predicted,\lambda }-R_{targeted,\lambda }\right)}^{2}\right]}^{1/2}$$

where the concentrations need to satisfy the constraint that *c*_*i*_ ≥ 0 and $\sum_{i=1}^{n}c_{i}=1$. It is notable that comparisons between these two types of optimization objectives have also been made (Sluban, 2007). To eliminate the metamerism phenomenon and achieve the unconditional match, spectrophotometric matching was adopted in this article. The constrained non-linear optimization algorithm, active-set algorithm, was applied to solve the spectrophotometric optimization objective.

Statistical results of the single-constant K-M theory for set A are shown in Table 1. It indicates that the tint used to determine the absorption and scattering ratio has a remarkable influence on the color prediction accuracy of the single-constant K-M theory. For all the tints, the maximum of the mean color difference reaches 3.5025 Δ*E*_00_, while the minimum is 1.4654 Δ*E*_00_, which is even better than that (1.5062) of the least square regression method; the maximum of the mean spectral error reaches 0.0882, while the minimum is 0.0423 which is very close to that (0.0421) of the least square regression method; the maximum of the mean CE reaches 0.1342, while the minimum is 0.0485, which is even better than that (0.0621) of the least square regression method. Moreover, the prediction accuracy is improved at first with the increase of the fractional concentration of the pre-colored fiber and then declines with the further increase of the fractional concentration. The optimal sample for the single-constant K-M theory can be selected as a tint with about 80% pre-colored fiber. This conclusion adheres to the inference drawn in the above section.

**TABLE 1:** Statistical results of single-constant K-M theory for Set A.

Fractional concentration of pre-colored fiber (%)	Δ *E*_00_		RMSE (%)		CE (%)	
	Max	Mean	Max	Mean	Max	Mean
20	6.8218	3.5025	0.1836	0.0882	0.2828	0.1060
40	5.6149	2.2917	0.1429	0.0559	0.2619	0.0599
60	4.0521	1.6587	0.1010	0.0423	0.1640	0.0485
80	3.5534	1.4654	0.1390	0.0449	0.1663	0.0815
100	5.1579	1.8859	0.2110	0.0693	0.2414	0.1342
Least square	2.9208	1.5062	0.1109	0.0421	0.1283	0.0621

Statistical results of the two-constant K-M theory for set A are shown in Table 2. Its average accuracy also shows that the prediction accuracy of the two-constant K-M theory is improved at first with the increase of the fractional concentration of the pre-colored fiber and then declines with the further increase of the concentration. The best performance of the two-constant K-M theory occurs when the tint has 40% pre-colored fiber. Its mean color difference is 0.1045 Δ*E*_00_, which is better than that (0.1307) of the least square regression method. Its mean spectral error is 0.0032, which is better than that (0.0041) of the least square regression method. Its mean CE is 0.0147, which is very close to that (0.0135) of the least square regression method. This phenomenon also adheres to the inference drawn in the above section. It means that the two samples, namely, a masstone and a tint mixed by 40% pre-colored fiber and 60% white fiber, are the best choice for the two-constant K-M theory.

**TABLE 2:** Statistical results of two-constant K-M theory for Set A.

Concentration of colored pigment (%)	Δ *E*_00_		RMSE (%)		CE (%)	
	Max	Mean	Max	Mean	Max	Mean
20	0.7644	0.1313	0.0179	0.0040	0.0600	0.0210
40	0.6419	0.1045	0.0162	0.0032	0.0399	0.0147
60	0.6043	0.1494	0.0199	0.0051	0.0461	0.0193
80	0.5375	0.1643	0.0187	0.0051	0.0536	0.0201
Least square	0.5759	0.1307	0.0163	0.0041	0.0350	0.0135

**FIGURE 4:**
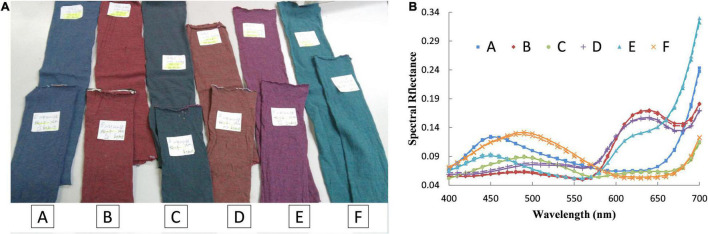
Six pairs of randomly selected matching samples. **(A)** The targeted samples (top) and predicted samples (bottom). **(B)** Spectral reflectance of targeted samples (solid lines) and predicted samples (dashed lines).

With a masstone and an optimal tint (mixed by 40% pre-colored fiber and 60% white fiber) for each pre-colored fiber, the two-constant K-M theory is implemented again on Set B to further verify the findings. Statistical results are collected in Table 3. It shows that the mean color difference of the optimal tint is 0.5367 Δ*E*_00_; the mean RMSE is 0.0102; and the mean CE is 0.0243. The performance is still better than that of the tint mixed by 60% pre-colored fiber and 40% white fiber. Thus, it can be concluded that the two samples, a masstone and a tint mixed by 40% pre-colored fiber and 60% white fiber, are enough to determine the absorption and scattering coefficients of a pre-colored fiber for the two-constant K-M theory. Six pairs of matching samples are randomly selected to intuitively test the results. As shown in Figure 4A, the color difference between the predicted and targeted samples is undistinguishable. Figure 4B shows the spectral reflectance of these samples. The spectral reflectance of each pair shows a high degree of coincidence.

**TABLE 3:** Statistical results of two-constant K-M theory for Set B.

Concentration of colored pigment (%)	Δ *E*_00_		RMSE (%)		CE (%)	
	Max	Mean	Max	Mean	Max	Mean
40	1.9919	0.5367	0.0251	0.0102	0.1244	0.0243
60	2.6699	0.6888	0.0326	0.0115	0.2219	0.0589

On the whole, two samples with certain fractional concentrations are enough for the two-constant K-M theory, and one sample is enough for the single-constant K-M theory. This can save sample preparation time, reduce the waste of resources and labor force, and increase productivity. On the contrary, the color prediction accuracy of the two-constant K-M theory is significantly better than that of the single-constant K-M theory for pre-colored fiber blends. It indicates that the optical characteristics of pre-colored fiber blends match the general assumptions that derive the two-constant K-M theory well, but fail to match the specific prerequisite for the derivation of the single-constant K-M theory. This result conforms to the above finding that the relationship between the optical characteristics and fractional concentrations of the two-constant K-M theory is more linear than that of the single-constant K-M theory. The two-constant K-M theory is more suitable for the color matching of the pre-colored fiber blends.

## Conclusion

The single-constant K-M theory and the two-constant K-M theory were examined to match the color of pre-colored fiber blends. The accuracy and the optimal samples used for the theories were evaluated based on the match results. It shows that the best sample for the single-constant K-M theory is a tint obtained by mixing 80% pre-colored fiber and 20% white fiber. A masstone obtained with 100% pre-colored fiber and a tint mixed by 40% pre-colored fiber and 60% white fiber are the best choice for the two-constant K-M theory. The findings can significantly reduce the samples required to be prepared in implementing the color matching, which can save time and raw materials for the companies. It also shows that due to the typical optical characteristics of the pre-colored fiber blends, the accuracy of the two-constant K-M theory is better than that of the single-constant K-M theory. The two-constant K-M theory is more suitable for the color matching of the pre-colored fiber blends. For further research, the applicability of the two-constant K-M theory for five or more pre-colored fiber blending needs to be investigated.

## Data Availability Statement

The original contributions presented in this study are included in the article/supplementary material, further inquiries can be directed to the corresponding author.

## Author Contributions

JL: methodology, data collection and analysis, and writing. DX: data collection, writing, and reviewing. ML: data collection. SL: data analysis. CW: methodology, funding acquisition, writing, and reviewing. All authors contributed to the article and approved the submitted version.

## Conflict of Interest

The authors declare that the research was conducted in the absence of any commercial or financial relationships that could be construed as a potential conflict of interest.

## Publisher’s Note

All claims expressed in this article are solely those of the authors and do not necessarily represent those of their affiliated organizations, or those of the publisher, the editors and the reviewers. Any product that may be evaluated in this article, or claim that may be made by its manufacturer, is not guaranteed or endorsed by the publisher.
